# Acute Cocaine Intoxication: An Approach to Severe Hepatic and Renal Dysfunctions

**DOI:** 10.7759/cureus.38524

**Published:** 2023-05-04

**Authors:** Pedro Fernandes Moura, Inês De Albuquerque Monteiro, Filipa S Pinho, Pedro M Neves, Pedro Silveira

**Affiliations:** 1 Internal Medicine, Centro Hospitalar do Médio Ave, Vila Nova de Famalicão, PRT; 2 Intensive Care, Hospital de Braga, Braga, PRT

**Keywords:** acute kidney failure, acetylcysteine, toxic hepatitis, cocaine toxicity, crack-cocaine

## Abstract

Cocaine is a highly addictive substance. Its poisoning can lead to potentially fatal multi-organ dysfunction. We report a case of cocaine overdose with severe multi-organ dysfunction.

A healthy 51-year-old man was admitted to the emergency room due to behaviour changes and seizure after inhaling crack. Multiple dysfunctions were developed, with emphasis on liver and kidney dysfunction, due to their severity. The patient had marked hepatic cytolysis with a peak on the third day with alanine aminotransferase (ALT) and aspartate aminotransferase (AST): 7941 and 4453 IU/L, respectively with mild coagulopathy and hyperbilirubinemia. Underwent empirical treatment with acetylcysteine ​​with good clinical response. Also developed anuric AKIN3 acute kidney injury secondary to rhabdomyolysis, requiring treatment with intermittent haemodialysis.

The approach to a case with severe multiorgan dysfunction is described, with special emphasis on the use of acetylcysteine. The good evolution of the patient can corroborate the use of this drug as a potential modifier of prognosis.

## Introduction

Cocaine, a benzathine acid, was originally used for its anaesthetic effects. However, its addictive properties perpetuated its misuse and turned it into a substance sought for recreational use. The scientific literature describes hyperthermia, arterial hypertension, tachycardia, liver cytolysis, kidney dysfunction, and multiorgan dysfunction, among others as possible manifestations of acute intoxication with this substance [1,2].

We present a case of acute cocaine intoxication with severe liver and kidney dysfunction requiring admission to the Intensive Care Unit.

## Case presentation

A 51-year-old man with chronic cocaine addiction, without other known medical conditions, developed behavioural changes with hetero aggressiveness and generalised tonic-clonic seizure after inhaling the drug. He was assisted by the pre-hospital emergency team and needed treatment with benzodiazepines. After stabilisation, he presented mild sedation, without focal neurological deficits. Was hemodynamically stable, without respiratory distress.

The cranioencephalic computed tomography (CT) showed no acute alterations. Analytically, presented 14490 leukocytes/μL (normal: 4000-10000/μL), 59000 platelets/μL (normal: 150000-400000/μL), creatinine 3.22 mg/dL (normal: 0.8-1.3 mg/dL), urea 74 mg/dL (normal: 10-50 mg/dL), myoglobin 167848 ng/mL (normal: 17-106 ng/mL), CK-total 34857 U/L (normal: 60-320 U/L), aspartate aminotransferase (AST) 1317 UI/L (normal: 10-34 UI/L), alanine aminotransferase (ALT) 304 UI/L (normal: 10-44 UI/L), international normalized ratio (INR) 1.76 (normal: 0.9-1.2), normal activated partial thromboplastin time (aPTT) and without hyperbilirubinemia or hypoalbuminemia. Also had troponin I: 8.195 ng/mL (normal: <0.03 ng/mL), although without angina or electrocardiographic changes. Urine drug abuse testing was positive for cocaine and negative for other drugs of abuse. Serum ethanol was negative.

The patient was admitted to an Intensive Care Unit due to acute cocaine intoxication with post-seizure crisis with rhabdomyolysis, associated with renal, hepatic, haematological, and cardiac dysfunction.

Hepatic dysfunction

**Figure 1:**
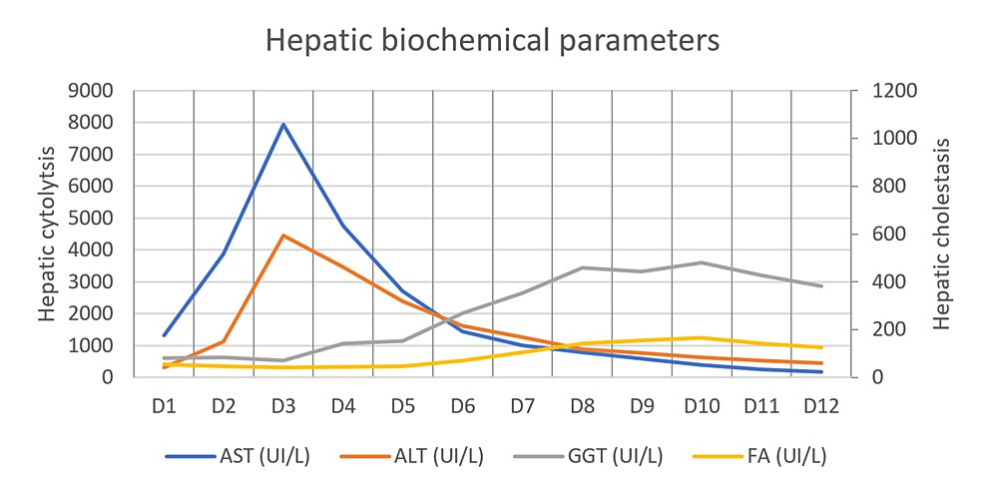
Evolution of hepatic profile AST: aspartate aminotransferase, ALT: alanine aminotransferase, GGT: gamma-glutamyl transferase, ALP: alkaline phosphatase, IU/L: International Units per Liter

**Figure 2:**
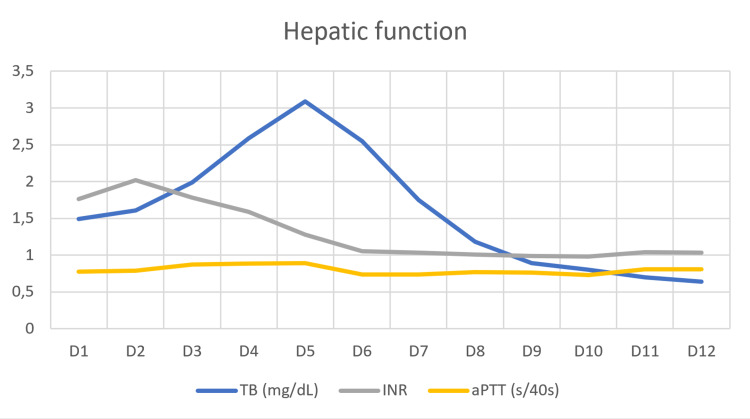
Evolution of serum bilirubin and coagulation study during hospitalization Total bilirubin (TB) in milligrams/decilitre (mg/dL) and activated partial thromboplastin time (aPTT) in seconds/40 seconds (s/40s)

A predominantly hepatocellular liver injury developed, with progressive aggravation peaking on the third day of hospitalisation: AST and ALT of 7941 and 4453 IU/L, respectively. This pattern was accompanied by liver dysfunction with elevated INR and aPTT peaking at 48h, with INR: 2.02 and aPTT: 31.4 seconds, and elevation of bilirubin, which peaked on the fifth day of hospitalisation, with total and direct bilirubin levels of 3.09 (normal: 0.20-1.00 mg/dL) and 2.07 mg/dL (normal: <0.30 mg/dL), respectively. Due to the severity of the clinical picture and the similarity to the pattern of acute paracetamol intoxication, despite the absence of evidence of paracetamol consumption, a protocol for paracetamol intoxication treatment with acetylcysteine was performed on the third and fourth day of hospitalisation. Throughout the remaining hospitalisation, there was a progressive overall improvement in liver dysfunction. Figure 1 and Figure 2 show the patient's evolution regarding liver function during hospitalisation. Other syndromes were excluded, and the remaining etiological study is attached in Table 1. Acute alcoholic hepatitis was ruled out. The patient had a single peak axillary temperature of 38°C upon admission, which prompted the collection of two sets of blood cultures for aerobic and anaerobic studies, without evidence of microbiological isolation. The patient remained afebrile during hospitalisation.

**Table 1:** Etiological study of liver dysfunction

Autoimmune pathology	
Antinuclear antibody (ANA)	Negative
Antineutrophil cytoplasmic antibodies (ANCA)	Negative
Smooth muscle antibodies	Negative
Anti-Liver kidney microsomal (Anti-LKM1) antibody	Negative
Antimitochondrial antibodies	Negative
Immunoglobulin G (IgG), Immunoglobulin M (IgM), Immunoglobulin A (IgA)	Within normal range values
Infectious disease	
Human immunodeficiency virus (HIV)	Anti-HIV I and II antibody: Negative
Hepatitis A virus (HAV)	HAV IgM: Negative
Hepatitis B virus (HBV)	Hepatitis B surface (HBs) antigen: Negative; Anti-HBs antibody: Negative; Anti-Hepatitis B core (Anti-HBc) antibody: Negative
Cytomegalovirus (CMV)	CMV IgG: Positive; CMV IgM: Negative
Epstein-Barr virus (EBV)	EBV IgM and EBV IgG: Negative
Herpes I and II	Herpes I and II IgG and IgM: Negative
Venereal Disease Research Laboratory (VDRL)	Non-reactive
Other pathologies	
Serum copper	Within normal range values
Abdominal computerized tomography (CT) scan and abdominal ultrasound with Doppler	Without relevant findings

Renal dysfunction

**Figure 3:**
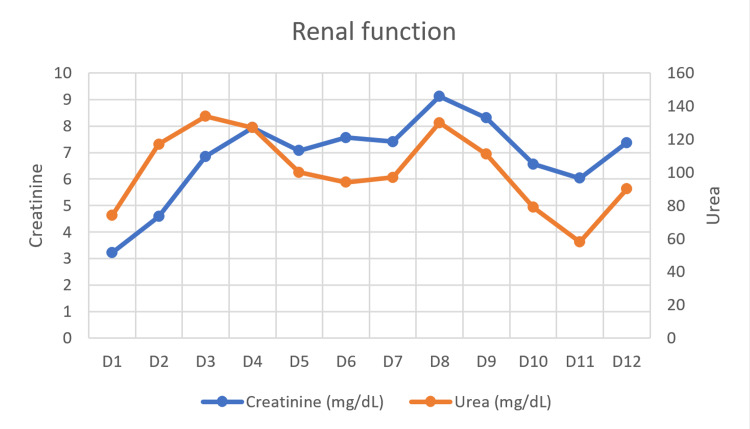
Evolution of renal function Serum creatinine and serum urea in milligrams/decilitre (mg/dL)

The patient developed anuric AKIN 3 acute kidney injury with active urinary sediment, without dysmorphic erythrocytes. The urinary protein/creatinine ratio was 4.1 mg/mg (normal: <0.15 mg/mg). Further study of renal dysfunction was performed and other etiologies of acute kidney injury were excluded (see Table 1 and Table 2). A myoglobinuric acute kidney injury was assumed, and urine alkalization was achieved by bicarbonate infusion. Despite medical treatment, there was progressive analytical deterioration, with sustained anuria, as shown in Figure 3. Intermittent renal replacement therapy was initiated on the third day of hospitalisation, and a total of seven sessions were performed daily. Urine output was eventually recovered with diuretic stimulation using furosemide and metolazone, and this treatment was gradually reduced throughout the remainder of the hospitalisation. At the time of discharge, the patient had serum creatinine and urea levels of 1.4 and 55 mg/dL, respectively.

Due to the improvement of all dysfunctions, the patient was transferred to the Internal Medicine ward on the 12th day of hospitalisation and was eventually discharged on the 32nd day with normalised renal function.

**Table 2:** Etiological study of renal dysfunction To be interpreted in conjunction with Table 1

Autoimmune pathology	
Anti-glomerular basement membrane (anti-GBM) antibody	Negative
Anti-double-stranded DNA (Anti-dsDNA) antibodies	Negative
Complement component 3 (C3) and Complement component 4 (C4)	Within normal range values
Urine light-chain proteins	Within normal range values
Cryoglobulin	Within normal range values
Other exams	
Renal ultrasound	Without relevant findings

## Discussion

Several mechanisms of cocaine-induced hepatotoxicity have been described in the literature. Some of them are direct hepatocellular damage due to toxic metabolites of cocaine, hyperthermia, and ischemia [1].

The manifestation of liver injury may arise hours to days after acute intoxication, presenting with liver dysfunction that may be accompanied by other organ dysfunctions. Analytically, hepatic toxicity in acute cocaine intoxication manifests as a disproportionate elevation of aminotransferases and lactate dehydrogenase (LDH) compared to alkaline phosphatase, with hyperbilirubinemia developing two to three days after consumption. These dysfunctions tend to resolve after one to two weeks [1].

In the presented case, the pattern of hepatocellular damage and timing of bilirubin elevation were consistent with those described in the literature. However, the unpredictability of these patients' behaviour does not allow us to exclude the role of toxicity from other potentially used adulterants.

The absence of a specific antidote for this drug and the progression to potentially compromising hepatic dysfunction supported by the similarity of the liver cytolysis pattern with that of acetaminophen intoxication led the medical team to administer acetylcysteine according to the protocol for acetaminophen intoxication [3].

Based on a direct association between the degree of transaminase elevation and mortality [4], and the good evolution of the patient after treatment with acetylcysteine, the favourable role of using this drug in intoxication whose exact mechanisms of hepatotoxicity are not yet fully understood is questioned. Despite mostly empirical use of the drug, cases of favourable responses to its use have been described [5]. In line with these findings, it is worth noting the report on the protective properties of acetylcysteine against cocaine-induced cellular toxicity [6].

The observed renal failure, with the need to initiate renal replacement therapy, seems to be explained by multifactorial injury, considering hypoperfusion/anoxic insult related to the convulsive episode and myoglobinuric injury.

In addition to these injury mechanisms, other mechanisms are also described as potentially implicated in nephropathy, among which are vasculitis secondary to the use of adulterants such as levamisole, renal infarction due to vasoconstriction or thrombosis from platelet adhesion, acute interstitial nephritis secondary to the drug or adulterants, thrombotic microangiopathy [7].

It is important to note that although this article focuses more on the approach to the hepatic and renal toxicity associated with this drug, other dysfunctions with therapeutic and prognostic impact were described in the case but not mentioned due to the favourable evolution.

## Conclusions

A case of acute cocaine intoxication with multiple associated dysfunctions is described. The literature reports a mortality rate of approximately 40% for episodes of acute liver failure, which is worse in cases of more severe dysfunction. Given the lack of evidence supporting any directed treatment, this report provides support for the beneficial prognostic role of acetylcysteine.

Understanding the pathophysiology of cocaine intoxication helps to guide the diagnostic and therapeutic approach to a syndrome with multi-organ involvement.
